# CD47-SIRP*α* Axis as a Biomarker and Therapeutic Target in Cancer: Current Perspectives and Future Challenges in Nonsmall Cell Lung Cancer

**DOI:** 10.1155/2020/9435030

**Published:** 2020-09-19

**Authors:** Rodrigo Catalán, Mario Orozco-Morales, Norma Y. Hernández-Pedro, Alberto Guijosa, Ana L. Colín-González, Federico Ávila-Moreno, Oscar Arrieta

**Affiliations:** ^1^Thoracic Oncology Unit, Instituto Nacional de Cancerología, Mexico City, Mexico; ^2^Laboratory of Personalized Medicine, Instituto Nacional de Cancerología, Mexico City, Mexico; ^3^Lung Diseases and Cancer Epigenomics Laboratory, Biomedicine Research Unit (UBIMED), Facultad de Estudios Superiores (FES) Iztacala, Universidad Nacional Autónoma de Mexico (UNAM), Mexico State, Mexico; ^4^Research Unit, Instituto Nacional de Enfermedades Respiratorias, Ismael Cosío Villegas, Mexico City, Mexico

## Abstract

CD47 is a cell surface protein in the immunoglobulin superfamily which is normally expressed at low levels in every healthy cell. It´s main physiologic function is to act as an inhibitor of phagocytosis; this occurs throughout interaction with SIRPa expressed on macrophages. Interaction between CD47 and SIRPa leads to activation of tyrosine phosphatases that inhibit myosin accumulation at the submembrane assembly site of the phagocytic synapse, resulting in phagocytosis blockade. In this way CD47 acts as a “don´t eat me signal” for healthy self-cells; accordingly, loss of CD47 leads to phagocytosis of aged or damaged cells. Taking advantage of this anti-phagocytic signal provided by CD47, many types of tumors overexpress this protein, thereby avoiding phagocytosis by macrophages and aiding in the survival of cancer cells. The aim of this review is to describe the physiologic the pathophysiologic role of CD47; summarize the available high-quality information about this molecule as a potential biomarker and/or therapeutic target in cancer; finally, we present an in-depth analysis of the available information about CD47 in association with nonsmall cell lung cancer, EGFR mutations, and tumor microenvironment.

## 1. CD47 Structure and Physiological Role

**Figure 1:**
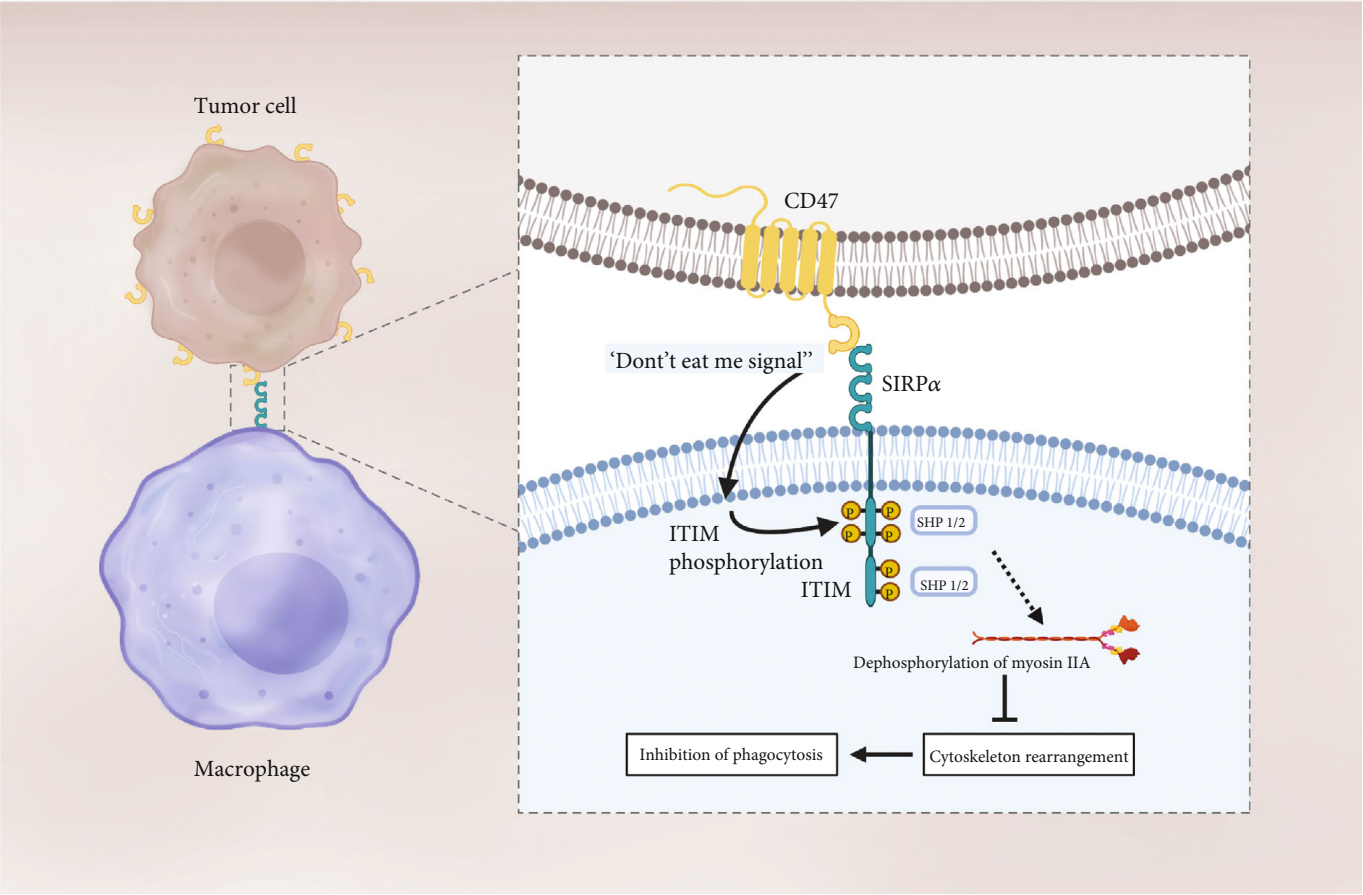
The CD47-SIRP*α* interaction plays a key role in phagocytosis inhibition. The CD47-SIRP*α* interaction leads to the phosphorylation of two tyrosine residues in the ITIM motif included in SIRP*α*'s cytosolic domain. Phosphorylation recruits and activates SHP1 & SHP2, signaling a cascade of events that leads to the dephosphorylation of myosin IIA and, therefore, inhibition of the cytoskeleton rearrangement, which is a necessary step for macrophages to engulf target cells. In tumor cells, the enhanced activity of the CD47-SIRP*α* axis is achieved by increasing CD47 expression.

CD47 is a transmembrane protein ubiquitously expressed in almost every healthy cell; it was first purified by Lindberg et al., in 1993 [1]. Structurally, it is a large protein composed of a long extracellular amino-terminal domain, five highly hydrophobic transmembrane domains, and a short hydrophilic carboxy-terminal cytoplasmic tail [2]. It has been demonstrated that the two principal ligands of CD47 are SIRP*α* and thrombospondin-1 (TSP-1) [3]. SIRP*α* is widely expressed on cellular surfaces of myeloid cells including several types of macrophages. Structurally, SIRP*α* has an extracellular immunoglobulin domain for ligand binding and a cytosolic domain that includes the immunoreceptor tyrosine-based inhibitory motif (ITIM), which enables its association with second-signalers (SHP1 &SHP2). Prior reports have demonstrated that interaction between extracellular CD47 and SIRP*α* results in the phosphorylation of two tyrosine residues in the intracellular ITIM domain of SIRP*α*. Afterwards, the phosphorylated tyrosines on SIRP*α* recruit and activate SHP1 and SHP2; this signaling cascade leads to the dephosphorylation of myosin IIA, therefore, inhibiting cytoskeleton rearrangement, which is a necessary step for macrophages to engulf target cells [4] (Figure 1).

The role of CD47 on immune recognition and phagocytosis was first described by Oldenborg et al., in 2000; their research demonstrated that healthy red blood cells (RBC) that are derived from *CD47^−/−^* mice were rapidly cleared when transfused to wild-type recipient mice; furthermore, they proved that this effect was reversed when macrophages were depleted using clodronate liposome [5]. Their results indicate that CD47 is one of the major components of red blood cells (RBCs) aging “clock,” which act by inhibiting erythrocyte phagocytosis until CD47 levels at RBCs membrane fall below the phagocytosis-inhibitory concentration, and RBCs are phagocyted by macrophages at the spleen [5]. Another pivotal experiment, which was published by Ishikawa-Sekigami et al., consisted in transferring normal RBC to mice lacking the ITIM domain of SIRP*α*; their results demonstrated that erythrocytes were rapidly phagocytosed, even when CD47 expression was high. This experiment confirmed that both components of the CD47-SIRP*α* axis play a key role in regulating phagocytosis of healthy cells [6].

Chao et al. were the first to propose that there must be a prophagocytic signal counterbalancing the effects of CD47 and providing an “eat me” signal to macrophages [7]. They also proposed that calreticulin (CRT) could be the molecule that provides this prophagocytic signal to macrophages. CRT is a highly conserved endoplasmic reticulum chaperone protein, which upon translocation from the endoplasmic reticulum to the cell surface provides an “eat me” signal; thereby promoting phagocytosis by macrophages and dendritic cells [8]. Apparently, it is the balance between antiphagocytic (i.e., CD47) and prophagocytic (i.e., CRT), which ultimately determines if a cell will be phagocyted or not [7–9].

The other main ligand of CD47 is TSP-1; this protein is a multidomain extracellular matrix glycoprotein, which binds to extracellular matrix components and cell surface receptors [10]. TSP-1 is mainly secreted by platelets and macrophages; binding of TSP-1 to CD47 leads to an increase in intracellular Ca^2+^, cAMP, and other second-signalers that regulate response to tissue damage, cell migration, and cell survival [3, 11]. Lacking a substantial cytoplasmic domain, information about proteins directly interacting with CD47 at the C-terminal intracellular domain is limited.

Almost every known intracellular downstream pathway of CD47 is controlled by TSP-1 binding; this does not exclude the possibility that SIRP*α* can also regulate CD47 downstream pathways. Rather, this merely reflects a general lack of experimental data; it should be noted that the effects of TSP-1 on CD-47 are beyond the scope of the present review.

## 2. The Role of CD47 in Cancer Pathophysiology

The pathogenesis of cancer depends on several hallmark features such as apoptosis evasion, sustained proliferation, angiogenesis, and immune system evasion [12]. The ability of cancer cells to evade and suppress the immune system was described over a century ago, and since then huge advances have been accomplished. Remarkably, the recognition of programmed cell death protein 1 (PD-1) and its ligand PD-L1, and the identification of their pathologic role in cancer, have led to the development of immune check-point inhibitors therapies, which have revolutionized the treatment of many solid tumors [13]. However, while adaptive immune response to tumors is well understood, and numerous drugs that improve adaptive immune response against solid tumors are available, the role of innate immunity was scantly studied until the last decade [14].

There are a lot of data suggesting that the innate immune response plays a fundamental role in modulating tumor phagocytosis through the CD47-SIRP*α* axis. CD47 was first identified as a tumor antigen on human ovarian cancer in the 1980s; since then, CD47 has been found to be overexpressed on multiple hematologic and nonhematologic malignancies, including chronic myeloid leukemia (CML), non-Hodgkin's lymphoma (NHL) [15], multiple myeloma [16], breast cancer [17], pancreatic cancer [18], nonsmall cell lung cancer (NSCLC) [19, 20], and other solid tumors. Increased expression of CD47 on tumors allows malignant cells to escape innate immune surveillance through evasion of phagocytosis by interacting with SIRP*α* on myeloid cells. In this way, cancer cells exploit the “don't eat me signal” provided by CD47. Of note, CD47 does not seem to be directly involved in regulating the proliferation and viability of cancer cells, as CD47-deficient cancer cells are phenotypically indistinguishable from their parental CD47 expressing cells in cell culture [4].

At the transcriptional level, several regulators of CD47 expression have been described. Stimulation of the tumor necrosis factor (TNF) pathway activates nuclear factor-*κ*B (NF-*κ*B) which directly binds to a constitutive enhancer of *CD47*, thereby increasing its transcription [21]. Hypoxia-inducible factor 1 (HIF-1) also binds to a *CD47* promoter, and there has been reported a strong correlation between HIF-1 and CD47 expressions [22]. Casey et al. described that MYC induces the transcription of both CD47 and PD-L1 in multiple tumors types, including lymphoma/leukemia and liver cancer [23].

## 3. CD47 As a Biomarker of Prognosis

Due to the commonly seen increased expression of CD47 in cancer cells, in the last decade, an increasing number of scientists have postulated CD47 as a prognostic factor in several types of tumors; however, evidence of the usefulness of CD47 as a biomarker has not been conclusive for every tumor. In 2009, Majeti et al. first reported that CD47 was a biomarker of poor prognosis in patients with acute myeloid leukemia (AML); in their superb publication, authors demonstrated that CD47 was more highly expressed on AML leukemia stem cells than their normal counterparts, and that increased CD47 expression predicted worse overall survival in three independent cohorts of adult AML patients [24].

The largest study in which CD47 has been reported as a biomarker of worse prognosis was presented by Zhang et al.; they demonstrated that increased CD47 mRNA expression levels were associated with a significantly decreased probability of overall survival in two independent datasets, which together comprised 1,954 patients with breast cancer. In the same publication, they reported that hypoxia-inducible factor 1 (HIF-1) directly activates the transcription of the CD47 gene in hypoxic breast cancer cells, and that knockdown of HIF activity or CD47 expression increased the phagocytosis of breast cancer cells by bone marrow-derived macrophages. In triple-negative breast cancer (TNBC), which is considered the most challenging breast tumor, increased CD47 expression was significantly associated with an advanced tumor-node-metastasis stage, lymph node involvement, and recurrence; authors also concluded that CD47 was an unfavorable and independent prognostic factor for 5-year disease-free survival in patients with TNBC [25]. The two previously mentioned studies, along with others [26, 27], have provided enough evidence to conclude that CD47 is a reliable biomarker of poor prognosis in patients with breast cancer. Another tumor in which CD47 has been consistently associated with poor prognosis is ovarian cancer; Li et al. reported that the higher CD47 expression was significantly correlated with poor prognosis of serous ovarian carcinoma patients. Furthermore, functional investigations revealed that the CD47 overexpression in ovarian cancer cells significantly promoted migration and invasion, and that CD47 induced epithelial-mesenchymal transition (EMT) through modulating E-cadherin and N-cadherin [28]. In a meta-analysis evaluating the prognostic significance of CD47 in human malignancies, which included 38 cohorts with a total of 7,229 patients, authors found that the increased expression of CD47 correlated with decreased overall survival (pooled HR = 1.49: 95% CI: 1.36-1.62, *p* < 0.001) in patients with six different types of tumors [29]. To the best of our knowledge, this was the first and only meta-analysis studying the prognostic significance of CD47 in several types of cancer, even if we consider this was a high-quality meta-analysis, there are some limitations while studying CD47 as a biomarker of prognosis in different types of cancer, other than hematologic and gynecologic (breast and ovarian) malignancies. First, there is no well-established detection method or cut-off value for evaluating the expression of CD47; while some groups utilize qPCR for quantification, others prefer using immunohistochemistry (IHC) and other DNA microarrays. Without a clear methodology and cut-off value for quantification (such as those widely used for PD-1/PD-L1), the prognostic value of CD47 will remain unclear in most malignancies. Second, CD47 expression may vary across populations; over half of the studies included in the aforementioned meta-analysis were performed in the Asian population; therefore, results should be validated in populations with different ethnicities.

## 4. CD47-SIRP*α* as a Therapeutic Target

Even if the signaling cascade of CD47 and its role as a biomarker remain incompletely understood, there is an increasing amount of evidence suggesting that the CD47-SIRP*α* axis is a potential therapeutic target for the treatment of several neoplasms. Accordingly, in the last decade, the targeting of CD47 has been in the spotlight of immunotherapy research.

There are several mechanisms by which the inhibition of the CD47-SIRP*α* axis can induce antitumor immunity. First, direct inhibition of CD47 or SIRP*α* promotes phagocytosis of tumor cells by disrupting the “don't eat me” signal provided by CD47, allowing the engulfment of tumor cells by macrophages. Second, an anti-CD47 antibody may eliminate tumor cells through Fc-dependent mechanisms, such as antibody-dependent cellular cytotoxicity (ADCC) and complement-dependent cytotoxicity (CDC). Third, analogous to what happens with macrophages, dendritic cells might also increase their phagocytic activity, leading to subsequent tumor antigen presentation to T cells and activation of the adaptive immune response [30]. Furthermore, there is evidence suggesting that CD47 might regulate apoptosis through a caspase-independent pathway [31, 32]. This last postulated mechanism (apoptosis induction) might be regulated by TSP-1 ligation to CD47; however, we consider that the underlying molecular mechanism regulating this process has not been well identified, and further studies are needed to corroborate if drugs targeting the CD47-SIRP*α* axis significantly promote apoptosis of tumor cells.

While the anti-CD47 antibody blocks a negative phagocytic signal, a positive phagocytic stimulus is still needed for phagocytosis. Accordingly, selective phagocytosis of malignant cells must be regulated by prophagocytic signals on the tumor. As mentioned earlier, tumors express the prophagocytic signal calreticulin (CRT) on the cell surface, while healthy counterparts usually do not express CRT, in this way, malignant cells which express high quantities of CRT will be much more susceptible to anti-CD47 mediated phagocytosis than their normal counterparts. However, it might still be possible that normal cells will also suffer from the other cytotoxic effects associated with blocking CD47, such as ADCC and CDC (discussed earlier) [7]. Whether or not blocking CD47 will produce widespread phagocytosis or cytotoxicity of healthy human cells remain unknown. However, as discussed later, it appears that targeting CD47 predominantly affects malignant cells and, to a lesser degree, erythrocytes.

Chao et al. were the first to explore *in vivo* if antibodies against CD47 could be a potential treatment for malignancies; astonishingly, they found that blocking anti-CD47 antibodies preferentially enabled phagocytosis of non-Hodgkin lymphoma (NHL) cells and synergized with rituximab (anti-CD20 antibody). In their study, treatment of human NHL-engrafted mice with anti-CD47 antibody reduced lymphoma burden and improved survival, while combination treatment with rituximab led to the elimination of lymphoma and cure [33]. This study was a pivotal achievement for translational oncology, and since then, several groups have explored the CD47-SIRP*α* axis as a therapeutic target in preclinical models [18, 34, 35].

In 2018, Advani et al. published the results of a phase 1b study involving 22 patients with relapsed or refractory NHL that were treated with Hu5F9-G4 (hereafter Magrolimab), which is a mAb against CD47, and rituximab (mAb against CD-20). The study reported promising results, with 50% of patients having an objective response (i.e., complete or partial response), and 36% achieving a complete response. Moreover, the combination proved to be safe, with few reported grades 3-4 adverse events, being the most common adverse effect anemia and infusion-related reactions [36]. This was the first result that demonstrated the clinical efficacy of targeting the CD47-SIRP*α* axis in humans. Later, in 2019, Sikic et al. published the result of a first-in-human, phase 1 clinical trial of Magrolimab (Gilead Sciences) in patients with advanced cancers, including solid tumors. The primary end of this point was to evaluate the safety, pharmacokinetics (PK), and pharmacodynamics (PD) of Magrolimab; in this study, 62 patients, which were heavily pretreated for their underlying malignancy before study enrollment (median of 5 previous systemic treatments; range one to 18), received Magrolimab at different doses. At the end of the study, no maximum tolerated dose was reached, and a priming dose of 1 mg/kg on day 1 followed by maintenance doses of up to 45 mg/kg weekly proved to be safe and was recommended for future studies [37]. In the aforementioned study, the most frequent adverse events were anemia, infusion-related reactions, and hemagglutination, all of them being mild to moderate in most patients. The two previously mentioned phase 1 trials definitely paved the way for other trials testing several drugs targeting the CD47-SIRP*α* axis.  Table 1 presents a list of drugs targeting the CD47-SIRP*α* axis, along or in combination with other therapies that are currently being tested in humans. Furthermore, there are several other drugs targeting this axis that are being tested at preclinical studies and hopefully will soon enter clinical trials in humans [3].

## 5. CD47- SIRP*α* in Nonsmall Cell Lung Cancer

Lung cancer is the leading cause of cancer-related death worldwide; among lung cancer subtypes, nonsmall cell lung cancer (NSCLC) is the most frequently diagnosed, accounting for 85% of newly diagnosed lung tumors. Even with the current state-of-the-art medical treatment, the prognosis for the overwhelming majority of NSCLC patients still remains poor [38]. Therefore, novel and efficient therapeutics for NSCLC are urgently needed [39].

In 2016, Zhao et al. were the first group to demonstrate that CD47 was overexpressed in NSCLC tumor tissues and cell lines, compared to matching adjacent nontumor tissues and normal bronchial epithelial cells, respectively. Moreover, the overexpression of CD47 significantly correlated with clinical staging, lymph node metastasis, and distant metastasis. On the cellular level, CD47 critically controlled the migration/invasion behavior of NSCLC cells, which was mediated through the regulation of Cdc42 expression. Nonetheless, the molecular mechanisms regulating Cdc42 remains poorly understood, and further research is warranted to understand the relationship between Cdc42 and CD47 [19]. Another study presented by Zhang el at. demonstrated that targeting CD47 by SIRP*α*–Fc mAb in NSCLC could elicit potent antitumor efficacy. During the treatment, autophagy was triggered via the inactivation of the Akt/mTOR signaling pathway and played a cytoprotective role in NSCLC cells. Simultaneously targeting CD47 and autophagy could elicit enhanced macrophage-mediated phagocytosis and cytotoxicity against NSCLC cells and showed enhanced inhibition or even complete elimination of NSCLC [40]. Later, this same group demonstrated that CD47 blockade could sensitize NSCLC to antiangiogenic therapy and potentiate its antitumor effects by enhancing macrophage infiltration and tumor cell destruction [41]. Taking into account the promising results obtained by combining anti-CD47 therapy with rituximab in NHL (discussed earlier), we consider the clinical trial testing if the combination of anti-CD47 therapy (such as Magrolimab) plus antiangiogenic therapy should begin in the foreseeable future and could provide a new therapeutic alternative for patients with NSCLC.

Recently, an *in vitro* study presented by Nigro et al. demonstrated that CD47 expression became upregulated in *EGFR* mutated NSCLC cell lines following the development of resistance to Gefitinib and blocking of CD47 by a specific mAb increased the clearance of *EGFR*-TKI resistant cells by phagocytes. Furthermore, on TKI-sensitive cell lines, EGFR inhibition significantly reduced CD47 expression on the surface of preapoptotic cells, favoring more efficient engulfment of cancer cells by monocyte-derived dendritic cells [20]. The results of these studies are on line with those recently reported by our group, which demonstrated that the presence of *EGFR* mutations and high expression of CD47 were associated with shortened PFS and OS [42]. However, the molecular pathway that modulates CD47 abundance in *EGFR*-mutated NSCLC remains incompletely understood and further research should be performed to determine if EGFR somehow regulates CD47. Of note, in the aforementioned study, our group did not identify CD47 overexpression as a prognostic factor for PFS and OS in NSCLC patients that did not harbor *EGFR* mutations. These results contrast with those reported by others, in which the high expression of CD47 was correlated with poor prognosis in patients with NSCLC.

In a retrospective study, Fucikova et al. reported that the level of CRT expression on tumor cells positively influenced the clinical outcome of NSCLC. High CRT expression on tumor cells was associated with a higher density of infiltrating mature dendritic cells and effector memory T-cells, suggesting that CRT might trigger the activation of adaptive immune responses in the tumor microenvironment [43].

## 6. Future Challenges for CD47-SIRP*α* Axis in Nonsmall Cell Lung Cancer

The role of CD47-SIRP*α* in NSCLC should be further studied in the basic and clinical settings. Basic research is needed to determine the precise molecular pathway that regulates CD47 expression, and to determine exactly how oncogenic mutations of *EGFR* are implicated in this regulation. It also remains to be fully understood whether or not CD47 can induce apoptosis by a caspase-independent pathway. Owing to opposing evidence, the role of CD47 and CRT, individually or in conjunction, as prognosis biomarkers in NSCLC should be studied prospectively to determine if these molecules are useful biomarkers.

**Figure 2:**
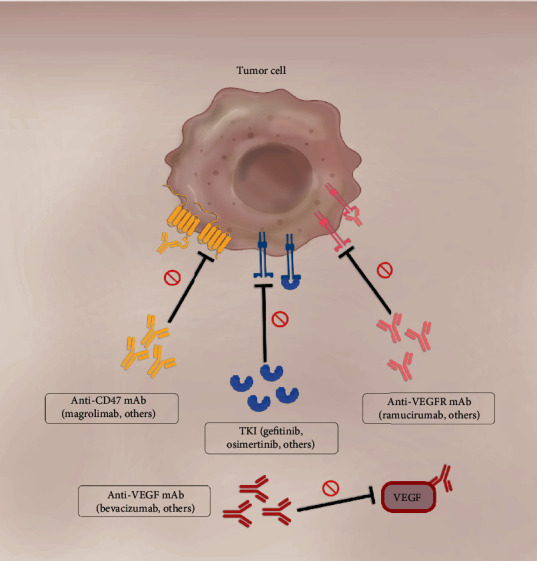
Promising treatment combinations of anti-CD47 drugs and current effective therapies for NSCLC. The inhibition of the CD47-SIRP*α* axis with anti-CD47 mAb can induce antitumor immunity through various mechanisms. Combining anti-CD47 with TKIs might be beneficial for two reasons. First, EGFR inhibition has shown to significantly reduce CD47 expression. Second, the inhibition of CD47 could increase the clearance of TKI-resistant cells. Combining anti-CD47 with antiangiogenic therapy could likewise be useful, since CD47 blockade could sensitize NSCLC to the latter therapy and potentiate its antitumor effects.

Finally, preclinical evidence suggests that CD47 inhibition might be a useful treatment strategy for patients with NSCLC; however, to the best of our current knowledge, there are currently no clinical trials specifically testing CD47 inhibitors in patients with NSCLC. In line with the evidence provided by treating NHL with Magrolimab plus rituximab, we consider that anti-CD47 drugs should be promptly tested in combination with other directed therapies, such as antiangiogenic therapy and tyrosine-kinase inhibitors, that have proved to be efficient for the treatment of NSCLC (Figure 2).

## 7. Conclusions

The CD47-SIRP*α* axis is at the spotlight of cancer immunotherapy research. Some relevant aspects regulating its activity remain to be understood; its usefulness as a biomarker also remains to be fully determined. Preclinical models allow us to hypothesize the usefulness of inhibiting this pathway in solid malignancies, such as NSCLC; we expect that in the near future, clinical trials will explore if combinations with other therapies could improve the prognosis of patients with several advanced malignancies, including NSCLC.

## Figures and Tables

**Table 1:** Drugs targeting CD47-SIRP*α* axis that are currently being tested in humans.

Drug name	Company	Mechanism of action	Clinical trial ID	Target malignancy	Combined with	Clinical phase	Status (June 2020)
Magrolimab (Hu5F-G4)	Gilead Sciences (previously, Forty-Seven Inc.)	Anti-CD47 mAb	NCT02953509	Relapsing NHL	Rituximab	Phase 1 & 2	Recruiting
			NCT03869190	Urothelial carcinoma	Several	Phase 1 & 2	Recruiting
			NCT03248479	AML & MDS	Azacitidine	Phase 1	Recruiting

BI735063	OSE Immunotherapeutics/Boehringer Ingelheim	Anti-SIRP*α* mAb	NCT03990233	Advanced solid tumors	Monotherapy or PD-1 inhibitor (BI 754091)	Phase 1	Recruiting

CC95251	Celgene	Anti- SIRP*α* mAb	NCT03783403	Advanced solid & hematologic cancers	Rituximab or Cetuximab	Phase 1	Recruiting

IBI188	Innovent Biologics (Suzhou) Co. Ltd.	Anti-CD47 mAb	NCT03763149	Advanced malignant tumors and lymphomas	Monotherapy	Phase 1	Recruiting
			NCT03717103	Advanced malignancies	Rituximab	Phase 1	Recruiting

TTI621	Trillium Therapeutics Inc.	SIRP*α*Fc-soluble recombinant fusion protein	NCT02663518	Hematologic malignancies and selected solid tumors	Rituximab or Nivolumab	Phase 1	Recruiting

TT622	Trillium Therapeutics Inc.	SIRP*α*-IgG4 Fc-soluble recombinant fusion protein	NCT03530683	Refractory lymphoma and myeloma	Monotherapy, rituximab, PD-1 inhibitor, or proteasome inhibitor	Phase 1	Recruiting

AO176	Arch Oncology	Anti-CD47 mAb	NCT03834948	Multiple solid malignancies	Monotherapy	Phase 1	Recruiting

ALX148	ALX Oncology Inc.	SIRP*α* soluble recombinant fusion protein	NCT03013218	Advanced Solid & Hematologic Cancers	Monotherapy, Pembrolizumab, Trastuzumab, rituximab, Ramucirumab+ Paclitaxel, 5-FU+ Cisplatin	Phase 1	Recruiting

## References

[1] Lindberg F. P., Lublin D. M., Telen M. J. (1994). Rh-related antigen CD47 is the signal-transducer integrin-associated protein. *The Journal of Biological Chemistry*.

[2] Brown E. J., Frazier W. A. (2001). Integrin-associated protein (CD47) and its ligands. *Trends in Cell Biology*.

[3] Zhang W., Huang Q., Xiao W. (2020). Advances in anti-tumor treatments targeting the CD47/SIRP*α* axis. *Frontiers in Immunology*.

[4] Feng M., Jiang W., Kim B. Y. S., Zhang C. C., Fu Y. X., Weissman I. L. (2019). Phagocytosis checkpoints as new targets for cancer immunotherapy. *Nature Reviews. Cancer*.

[5] Oldenborg P. A., Zheleznyak A., Fang Y. F., Lagenaur C. F., Gresham H. D., Lindberg F. P. (2000). Role of CD47 as a marker of self on red blood cells. *Science*.

[6] Ishikawa-Sekigami T., Kaneko Y., Saito Y. (2006). Enhanced phagocytosis of CD47-deficient red blood cells by splenic macrophages requires SHPS-1. *Biochemical and Biophysical Research Communications*.

[7] Chao M. P., Jaiswal S., Weissman-Tsukamoto R. (2010). Calreticulin is the dominant pro-phagocytic signal on multiple human cancers and is counterbalanced by CD47. *Science Translational Medicine*.

[8] Obeid M., Tesniere A., Panaretakis T. (2007). Ecto-calreticulin in immunogenic chemotherapy. *Immunological Reviews*.

[9] Feng M., Marjon K. D., Zhu F. (2018). Programmed cell removal by calreticulin in tissue homeostasis and cancer. *Nature Communications*.

[10] Koduru S. V., Sun B. H., Walker J. M. (2018). The contribution of cross-talk between the cell-surface proteins CD36 and CD47-TSP-1 in osteoclast formation and function. *The Journal of Biological Chemistry*.

[11] Soto-Pantoja D. R., Kaur S., Roberts D. D. (2015). CD47 signaling pathways controlling cellular differentiation and responses to stress. *Critical Reviews in Biochemistry and Molecular Biology*.

[12] Hanahan D., Weinberg R. A. (2011). Hallmarks of cancer: the next generation. *Cell*.

[13] Chen D. S., Mellman I. (2017). Elements of cancer immunity and the cancer-immune set point. *Nature*.

[14] Jaiswal S., Chao M. P., Majeti R., Weissman I. L. (2010). Macrophages as mediators of tumor immunosurveillance. *Trends in Immunology*.

[15] Chao M. P., Tang C., Pachynski R. K., Chin R., Majeti R., Weissman I. L. (2011). Extranodal dissemination of non-Hodgkin lymphoma requires CD47 and is inhibited by anti-CD47 antibody therapy. *Blood*.

[16] Kim D., Wang J., Willingham S. B., Martin R., Wernig G., Weissman I. L. (2012). Anti-CD47 antibodies promote phagocytosis and inhibit the growth of human myeloma cells. *Leukemia*.

[17] Willingham S. B., Volkmer J. P., Gentles A. J. (2012). The CD47-signal regulatory protein alpha (SIRPa) interaction is a therapeutic target for human solid tumors. *Proceedings of the National Academy of Sciences of the United States of America*.

[18] Michaels A. D., Newhook T. E., Adair S. J. (2018). CD47 blockade as an adjuvant immunotherapy for resectable pancreatic cancer. *Clinical Cancer Research*.

[19] Zhao H., Wang J., Kong X. (2016). CD47 promotes tumor invasion and metastasis in non-small cell lung cancer. *Scientific Reports*.

[20] Nigro A., Ricciardi L., Salvato I. (2020). Enhanced expression of CD47 is associated with off-target resistance to tyrosine kinase inhibitor gefitinib in NSCLC. *Frontiers in Immunology*.

[21] Betancur P. A., Abraham B. J., Yiu Y. Y. (2017). A CD47-associated super-enhancer links pro-inflammatory signalling to CD47 upregulation in breast cancer. *Nature Communications*.

[22] Zhang H., Lu H., Xiang L. (2015). HIF-1 regulates CD47 expression in breast cancer cells to promote evasion of phagocytosis and maintenance of cancer stem cells. *Proceedings of the National Academy of Sciences of the United States of America*.

[23] Casey S. C., Baylot V., Felsher D. W. (2018). The MYC oncogene is a global regulator of the immune response. *Blood*.

[24] Majeti R., Chao M. P., Alizadeh A. A. (2009). CD47 is an adverse prognostic factor and therapeutic antibody target on human acute myeloid leukemia stem cells. *Cell*.

[25] Yuan J., Shi X., Chen C. (2019). High expression of CD47 in triple negative breast cancer is associated with epithelial-mesenchymal transition and poor prognosis. *Oncology Letters*.

[26] Yuan J., He H., Chen C., Wu J., Rao J., Yan H. (2019). Combined high expression of CD47 and CD68 is a novel prognostic factor for breast cancer patients. *Cancer Cell International*.

[27] Baccelli I., Stenzinger A., Vogel V. (2014). Co-expression of MET and CD47 is a novel prognosticator for survival of luminal-type breast cancer patients. *Oncotarget*.

[28] Li Y., Lu S., Xu Y. (2017). Overexpression of CD47 predicts poor prognosis and promotes cancer cell invasion in high-grade serous ovarian carcinoma. *American Journal of Translational Research*.

[29] Zhao H. J., Pan F., Shi Y. C. (2018). Prognostic significance of CD47 in human malignancies: a systematic review and meta-analysis. *Translational Cancer Research*.

[30] Chao M. P., Weissman I. L., Majeti R. (2012). The CD47-SIRP*α* pathway in cancer immune evasion and potential therapeutic implications. *Current Opinion in Immunology*.

[31] Mateo V., Lagneaux L., Bron D. (1999). CD47 ligation induces caspase-independent cell death in chronic lymphocytic leukemia. *Nature Medicine*.

[32] Manna P. P., Frazier W. A. (2004). CD47 mediates killing of breast tumor cells via Gi-dependent inhibition of protein kinase A. *Cancer Research*.

[33] Chao M. P., Alizadeh A. A., Tang C. (2010). Anti-CD47 antibody synergizes with rituximab to promote phagocytosis and eradicate non-Hodgkin lymphoma. *Cell*.

[34] Tzatzarakis E., Hissa B., Reissfelder C., Schölch S. (2019). The overall potential of CD47 in cancer immunotherapy: with a focus on gastrointestinal tumors. *Expert Review of Anticancer Therapy*.

[35] Huang Y., Ma Y., Gao P., Yao Z. (2017). Targeting CD47: the achievements and concerns of current studies on cancer immunotherapy. *Journal of Thoracic Disease*.

[36] Advani R., Flinn I., Popplewell L. (2018). CD47 blockade by Hu5F9-G4 and rituximab in non-Hodgkin’s lymphoma. *The New England Journal of Medicine*.

[37] Sikic B. I., Lakhani N., Patnaik A. (2019). First-in-human, first-in-class phase i trial of the anti-CD47 antibody Hu5F9-G4 in patients with advanced cancers. *Journal of Clinical Oncology*.

[38] Bray F., Ferlay J., Soerjomataram I., Siegel R. L., Torre L. A., Jemal A. (2018). Global cancer statistics 2018: GLOBOCAN estimates of incidence and mortality worldwide for 36 cancers in 185 countries. *CA: A Cancer Journal for Clinicians*.

[39] Zhang C., Leighl N. B., Wu Y. L., Zhong W. Z. (2019). Emerging therapies for non-small cell lung cancer. *Journal of Hematology & Oncology*.

[40] Zhang X., Fan J., Wang S. (2017). Targeting CD47 and autophagy elicited enhanced antitumor effects in non-small cell lung cancer. *Cancer Immunology Research*.

[41] Zhang X., Wang Y., Fan J. (2019). Blocking CD47 efficiently potentiated therapeutic effects of anti-angiogenic therapy in non-small cell lung cancer. *Journal for Immunotherapy of Cancer*.

[42] Arrieta O., Aviles-Salas A., Orozco-Morales M. (2020). Association between CD47 expression, clinical characteristics and prognosis in patients with advanced non-small cell lung cancer. *Cancer Medicine*.

[43] Fucikova J., Becht E., Iribarren K. (2016). Calreticulin expression in human non-small cell lung cancers correlates with increased accumulation of antitumor immune cells and favorable prognosis. *Cancer Research*.

